# Toitū te Tiriti: A Tiriti o Waitangi-led Approach to Public Health Curriculum Development

**DOI:** 10.1177/15248399231163565

**Published:** 2023-03-22

**Authors:** Christina Severinsen, Bevan Erueti, Linda Murray, Suzanne Phibbs, Christine Roseveare, Charles Egwuba

**Affiliations:** 1 Massey University, Palmerston North, New Zealand

**Keywords:** health promotion, Indigenous, decolonization, professional preparation, pedagogy, continuing education, curriculum, cultural competence, Māori, workforce development

## Abstract

At Te Kunenga ki Pūrehuroa (Massey University), Aotearoa New Zealand, we have declared our stance as a Te Tiriti o Waitangi-led institution. This necessitates the embodiment and enactment of the principles and provisions of Te Tiriti o Waitangi and the embedding of Indigenous Māori knowledge, values and belief systems in curriculum design and implementation. This article outlines the beginning of our journey toward indigenizing our postgraduate public health curriculum at Te Kunenga ki Pūrehuroa. We describe the redevelopment of the Master of Public Health curriculum that embeds mātauranga Māori (Māori knowledge), te reo Māori (Māori language), tikanga Māori (Māori values and belief systems), and Māori pedagogy (culturally sustaining teaching and learning practices). Here, we focus on how curriculum redevelopment and pedagogy have enabled the utility of Māori knowledge and processes to be reflected at every level of the program and give life and relevancy to Te Tiriti o Waitangi. Te Tiriti o Waitangi guides our teaching practice and ensures that students can safely develop their confidence in Māori ways of knowing, being, and doing to effectively partner with Māori as Tangata Whenua. Our program aims to produce agentic graduates who are champions and advocates for Māori aspirations in health.

## Health promotion, Indigenous Knowledge, and Te Tiriti O Waitangi

Internationally, it is recognized that health promoters must have the skills to appraise the effectiveness of past and current public health work in light of continuing inequitable health outcomes and critically reflect on their discipline’s history. In Aotearoa NZ, health promotion has a *whakapapa* (see Table 1) in social justice and equity. Health promotion action must align with *Te Tiriti o Waitangi* aspirations of relationships, *rangatiratanga*, and equity (Berghan et al., 2017; Severinsen et al., 2021). Health promoters must be critically aware of the ongoing effects of colonization, be competent in applying *Te Tiriti o Waitangi* in their practice and engage with Māori communities (Berghan et al., 2017; Health Promotion Forum, 2012; Severinsen et al., 2021). As a *Te Tiriti o Waitangi*-led institution, we at *Te Kunenga ki Pūrehuroa*, Massey University, deem it necessary to embody and enact the principles and provisions of *Te Tiriti*. By prioritizing Te Tiriti as a living document, we are required to “promote the determination of Māori-led aspirations, active use of Te Reo Māori and the vitality and well-being of all people and our environment to give full and authentic expression to the eminence of Te Tiriti o Waitangi” (Massey University, 2018).

**Table 1:** Glossary of Terms

Ākonga	Students
Hapū	Sub tribe
Hauora	Well-being
Iwi	Tribe
Kaupapa Māori	A Māori way
Māori	Indigenous population of Aotearoa New Zealand
Manaakitanga	Generosity and caring for others
Mātauranga Māori	Indigenous Māori knowledge, values and belief systems
Mihimihi	Speech of greeting
Pepeha	Introducing oneself in Te Reo Māori, sharing connections with people and place
Rangatiratanga	Māori self-determination, sovereignty
Tangata Whenua	Indigenous Māori, people born of the land
Te Kunenga ki Pūrehuroa	A metaphorical name translated as “From inception to infinity” gifted by Kaumātua (elder) Kāhu Stirling, referring to the idea that learning is never-ending
Te Tiriti o Waitangi	Māori (the Indigenous peoples) have a national treaty negotiated with the colonizing British Crown and signed in 1840. The treaty, Te Tiriti o Waitangi, guarantees continued Māori sovereignty, protects Māori interests, promotes Māori well-being, and guaranteed the Crown limited kāwanatanga (governance).
Tikanga Māori	Correct procedures, customs, protocols
Wānanga	To meet and discuss
Whakamana	Empowerment and legitimacy of those involved, giving effect to, enacting
Whakamārama	Enlightenment, process of illuminating and explaining, building clarity in knowledge and understanding
Whakapapa	Geneology, lineage
Whakapiri	Engagement, building close connections, or relationships, between people
Whānau	Extended family group
Whanaungatanga	Building relationships

## Te Tiriti-led Public Health Curriculum Development

In curriculum design and implementation, being *Te Tiriti*-led is an approach that re-centers *mātauranga Māori*. The co-design of our Master of Public Health program was carried out through Te Ohu Manaaki. This advisory group was built from existing *whanaungatanga* with Māori academic staff within the program, our colleagues from Te Pūtahi-a-Toi (School of Māori Knowledge) and the wider university, and external Māori public health organizations, academics, and sector bodies. The process of curriculum development was informed by *kaupapa Māori*, *tikanga Māori*, and *manaakitanga*. Discussion in this space maintained a *Te Tiriti o Waitangi* lens that privileged Māori voices and rangatiratanga to ensure the curriculum reflects the aspirations of Māori communities. For example, the co-construction of the graduate profile and collective mapping of the Aotearoa NZ Public Health competencies framework with course learning outcomes created a shared collective vision for the program through consensus decision-making and collaboration. The curriculum focuses on the social determinants of health, and *whānau*-led health promotion approaches to ensure that graduates are confident to work with Māori communities and contribute to advanced health outcomes for whānau, hapū and iwi. Hauora Māori and Te Tiriti o Waitangi were absent in the program prior to redevelopment, and are now embedded at every program level, reflected in the graduate profile and in the learning outcomes for every course. Ongoing collaborative development and delivery of the program with Māori academics and Māori public health experts will ensure the relevance and quality of the curriculum for improving the health of Māori communities.

To guide this approach is a Māori-centric university-wide teaching and learning strategy called Paerangi (Massey University, 2019). Developed by Māori pedagogical experts, higher education experts and linguists within Te Kunenga ki Pūrehuroa and externally, and complemented by an inclusive and robust consultation process, Paerangi sets out our values, approaches, and practices toward learning and teaching that reflect the episteme of Māori knowledge. It invites all teaching staff to enact several Māori concepts that give tangibility to the integration of a Māori perspective of “best educational practice” that we believe not only enhances the learning experience of ākonga Māori but also non-Māori students.

## Te Tiriti Aligned Pedagogy

The Master of Public Health program aims to give effect to what it means to be Te Tiriti-led through our curricula and pedagogies. First, reflexivity and relationality are core to our teaching practice. Health promoters should hold sociocultural knowledge of the place they are of and for. Tangata whenuatanga, place-based learning, requires students to first connect themselves within Aotearoa NZ. Embedding learning about settler colonialism and *Te Tiriti o Waitangi* opens spaces to discuss and reflect on the context of health outcomes. Critical pedagogies of place ask us to engage as citizens in our place and consider how we can contribute to the well-being of people and places (Severinsen et al., 2020). For non-Māori, it means “becoming more competent at understanding and being who we are now, here, in Aotearoa” (Hoskins & Jones, 2022). We provide opportunities for students to reflect on what it means to be a health promoter in Aotearoa NZ, and how they will be meaningful and relevant in their contribution to public health both nationally and globally. For example, our students engage in experiential learning praxis courses and *wānanga*. All students have the opportunity for applied learning through community and civic engagement, enabling them to connect with the broader community actively and authenticate their study through real-world application. Our course 231704 *Hauora Māori* offers an in-depth, critical exploration of Indigenous health beyond a deficit focus on inequity and poor health outcomes to cultural strengths and Indigenous ways of knowing. Students engage in a positionality exercise, incorporating *mihimihi* and *pepeha*, and critically reflect on their own understandings and engagement with the principles and provisions of *Te Tiriti*.

This relational approach is also reflected in the utilization of the *Whakapiri*, *Whakamārama*, and *Whakamana* (Durie, 2008) framework. This relational and processional model outlines the achievement of learning outcomes at both the whole program and course levels. Our *ākonga* journey through a progression that begins with their engagement (Whakapiri), encourages their thinking into enlightenment (Whakamārama), and advances their criticality into empowerment (Whakamana). We ensure that all courses, pedagogies, and supporting infrastructure are designed to enable active and participatory learning and develop students’ critical thinking skills via this framework. The Master of Public Health aims to build reflective practice skills with students, providing integrated opportunities in coursework to hone the critical thinking required for public health practice. We aim to empower students to be active partners in their learning by fostering an engagement with the learning process alongside disciplinary knowledge, providing meaningful feedback opportunities to improve and enhance learning. Portfolio assessment woven through the program provides a solid foundation for reflective practice, iterative learning processes, and reciprocal learning between students and staff.

## Conclusion

The challenges of teaching Indigenous health within largely Eurocentric domains risk placing Indigenous content on the margins (Ahuriri-Driscoll et al., 2021; Coombe et al., 2017) or reinforcing dominant deficit approaches to Indigenous health. Strong calls have been made which promote the careful design of curricula for decolonizing and Indigenizing public health. The IUHPE Waiora statement calls for health promotion “to make space for and privilege Indigenous peoples’ voices and Indigenous knowledge in promoting planetary health and sustainable development for the benefit of all.” In curriculum design, this includes horizontal and vertical integration of Indigenous knowledge across programs (Coombe et al., 2017, 2019; Lee et al., 2017). In support of such calls, we, at the time of writing this description of *Te Tiriti* pedagogical responsiveness and practice within our programs, were attending several *wānanga* (each lasting 3 days) to accelerate a pan-Massey University approach to *Te Tiriti* provision, integration and delivery in all areas of the university. The first of these *wānanga* invited each participant, as non-Māori or as *Tangata Whenua*, to position themselves as *Te Tiriti* partners. These shared experiences and goals enable us to drive “inclusion of Indigenous knowledge, cultural responsiveness, preparing students to work effectively with Indigenous communities, a focus on equity, and consideration of institutional racism” (Ahuriri-Driscoll et al., 2021, p. 150). It is apparent that robust relationships are foundational to this work of decolonization and the creation of agentic change. This takes time, and a major systemic challenge of working in a “mainstream” educational institution is the continual undermining of “time” for relationship-building strategies that are necessary powerful drivers of our teaching and learning. By taking the time, we feel affirmed in our enactment of working toward the Indigenization of our public health curriculum that upholds the place of Māori as *Tangata Whenua*, especially where there is a positive impact on *ākonga Māori*.
